# The Genetic Background of Endometriosis: Can ESR2 and CYP19A1 Genes Be a Potential Risk Factor for Its Development?

**DOI:** 10.3390/ijms21218235

**Published:** 2020-11-03

**Authors:** Beata Smolarz, Krzysztof Szyłło, Hanna Romanowicz

**Affiliations:** 1 Laboratory of Cancer Genetics, Department of Pathology, Polish Mother’s Memorial Hospital Research Institute, Rzgowska 281/289, 93-338 Lodz, Poland; hanna-romanowicz@wp.pl; 2 Department of Operative Gynaecology and Oncological Gynaecology, Polish Mother’s Memorial Hospital Research Institute, Rzgowska 281/289, 93-338 Lodz, Poland; kszyllo@o2.pl

**Keywords:** endometriosis, genes, ESR2, CYP19A1, polymorphism, expression

## Abstract

Endometriosis is defined as the presence of endometrial foci, localized beyond their primary site, i.e., the uterine cavity. The etiology of this disease is rather complex. Its development is supported by hormonal, immunological, and environmental factors. During recent years, particular attention has been focused on the genetic mechanisms that may be of particular significance for the increased incidence rates of endometriosis. According to most recent studies, ESR2 and CYP19A1 genes may account for the potential risk factors of infertility associated with endometriosis. The paper presents a thorough review of the latest reports and data concerning the genetic background of the risk for endometriosis development.

## 1. Introduction

Endometriosis is a medical condition characterized by the presence of active foci of uterine mucous membrane (glandular and stromal cells) or of endometrial tissue cells (endometrioides, where *eides* means “similar” in Greek) localized beyond the uterine cavity, i.e., in the muscular layer of the uterus as well as in other genital organs and at their regions, and even at other places of the body, distant from the genital organs [1]. Off-uterine endometrial foci may, for example, occur in the peritoneal cavity, the ovaries, the uterine bladder, or in the ureters [2]. The ectopic endometrium demonstrates functional similarity to the eutopic mucous membrane of the uterus.

Endometriosis is a mild, estrogen-dependent, gynecological condition; however, taking into account its chronic character and accompanying complaints, it is approached as a major medical, social, and economic problem.

Endometriosis is a frequent gynecological condition both in Poland and in the world. This disease affects 10–15% of women in the reproductive age and 35–50% of women with pains in the pelvis minor and/or with infertility. One should keep in mind, however, that sporadic cases of endometriosis are also diagnosed in patients after menopause, as well as in young women [3].

The vast majority of endometriosis cases are diagnosed in women between menarche and menopause. The maximum incidence of endometriosis is observed between the 25th and the 45th years of life [4]. 

According to literature data, endometriosis is confirmed in 0.1–53% of women operated by laparoscopy or laparotomy [5,6], of whom 12–32% are women after diagnostic laparoscopy, conducted for chronic pain within the pelvis minor, and 10–60% are patients after diagnostic laparoscopy, performed for infertility. 

In 7% of women, endometriosis is associated with its familial history. The disease has been identified in 2% of women after ovarian sterilization and in 17% of women after ovariectomy [4,7].

Some world literature reports also present cases where endometriosis foci occurred in fetuses [8].

Single cases of endometriosis have been diagnosed in the world in men after a hormonal therapy for prostate cancer [9].

The risk of contracting endometriosis is the lowest in black women and the highest in women of the mongoloid race. Women of the white race demonstrate a higher risk for endometriosis than black women [10].

Endometriosis is a large-scale problem not only in medical and social approach but also from the economic point of view. The annual costs of endometriosis treatment in Europe vary from 0.0 to 12.5 billion EURO, depending on countries, and are comparable with the costs of therapy of other chronic diseases, e.g., diabetes mellitus [11].

Endometriosis exerts a substantial negative impact on many aspects of social, family, sexual, educational, and occupational activity as well as its adverse impacts may also be found in daily life [11,12,13]. Pain and the associated systemic dysfunction deteriorate the quality of life and reduce the occupational productivity. In cases with unclear etiology or where medical treatment is not available, the disease may be chronic and recurring. Considering its effects on sexuality and fertility, it may have negative consequences for relations in partnerships.

## 2. The Genetic Background of Endometriosis

Despite numerous studies on endometriosis markers, there have still been no satisfying results, which precludes effective laboratory diagnostics, which is employed to identify the disease and monitor its treatment [14].

### 2.1. MicroRNA (MiRNA)

Studies of blood-circulating microRNAs [15] provide certain grounds for justified expectations of endometriosis marker discovery. MicroRNAs are small particles of ribonucleic acid with their length of about 22 nucleotides. They control the expression of genes, exerting an influence on the translation process [16]. MicroRNAs demonstrate stability in tissue and are easily detectable in serum from patients by quantitative methods, such as qPCR. A successful assay of a single microRNA may help distinguish a healthy person from one with the disease. However, only the assay of several microRNAs, the expression of which changes in a given disease, presents the highest diagnostic strength. 

The to-date studies of microRNAs in endometriosis have proven that a certain group of genes is controlled with participation of short, relatively stable RNA fragments. The microRNAs of the *let* family belong to dominating ones in endometrial cells. It has been demonstrated in the study by Sahin et al. that the Let-7b microRNA affects the expression of *ER-α*, *ER-ß*, *Cyp19*, *KRAS 4A*, *KRAS 4B,* and *IL-6* genes. Let-7b reveals pleiotropic activity in the pathophysiology of endometriosis, affecting the control of the levels of estrogen and growth factor receptors [17].

Liu et al. suggested that the expression of miR‑449b‑3p changed in endometriosis and supported the development of the disease [18]. 

Changed expression levels of miR-139-5p and miR-375 have been observed in the tissues of ectopic endometrium. The abovementioned microRNAs may control the expression of *HOXA9* and *HOXA10* transcriptive factors and of endothelin-1 gene (*EDN1*), playing some role in vascular homeostasis, which may be associated with the development of endometriosis [19].

The studies of Laudanski et al. have shown that the genes of the mammalian target of rapamycin and of the vascular endothelial growth factor (VEGF) may be controlled by an abnormal expression of miRNA in endometriosis [20]. The mTOR (mammalian target of rapamycin kinase) kinase controls the processes of growth, proliferation, and mobility of cells, as well as translation and transcription processes, while VEGF enhances the process of angiogenesis in endometriosis. A team of Polish researchers conducted miRNA profiling in eutopic endometrium samples from women with endometriosis. They studied 667 human miRNAs in patients with endometriosis and compared the obtained results with those in a control group. Two miRNAs from the studied pool demonstrated an increased expression, while 13 other miRNAs were characterized by decreased expression levels in eutopic endometrium in patients with endometriosis, when compared to the control group. It was also demonstrated that hsa-miR-483-5p and miR-629 were characterized by a considerably reduced expression in patients with endometriosis [21]. 

Studies of the pool with different miRNAs in endometriosis have shown that many biological processes are controlled by the particles, which may have a significant effect for the development of pathological changes (Table 1). 

The use of microRNAs in the diagnostics of endometriosis is, however, only in the preliminary stage of studies [22,23]. 

### 2.2. Long Noncoding RNA (lncRNA)

Long noncoding RNAs (lncRNAs) have recently become the object of interest in the context of endometriosis. It concerns the lncRNAs that encompass more than 200 bp and are a subtype of noncoding RNAs (ncRNAs) [24].

Unlike the group of short noncoding RNAs (sncRNAs), such as microRNA, long noncoding RNAs (lncRNAs) usually demonstrate a better match of sequences, i.e., a higher specificity of their activity with regards to target genes. The noncoding RNA molecules participate in regulation processes, practically at all the stages of genetic information transmission: From DNA to protein. Especially spectacular is the involvement of some noncoding RNA molecules in the mechanisms, leading to activation or inactivation of the expression of particular genes.

In the studies by Zhou et al., 388 examined lncRNA transcripts demonstrated overexpression, while 188 presented with reduced expression levels in ectopic endometrium vs. eutopic endometrium [24]. 

It is known that the expression of many lncRNAs undergoes changes in (1) blood serum of women with endometriosis vs. healthy women, (2) in ectopic endometrium of the ovaries in comparison with eutopic endometrium of women with endometriosis, and (3) eutopic endometrium of women with endometriosis vs. healthy women [25]. 

The types of lncRNAs and their expression changes in endometriosis are presented by Table 2. Nevertheless, the clinical significance and the biological mechanism of lncRNA in the development of endometriosis remain largely unknown.

### 2.3. Genome-Wide Association Studies (GWAS)

The epidemiology of endometriosis may better be learned via studies on the human genome, especially supported by GWAS development, i.e., association studies of the entire genome. 

The initial GWAS projects, concerning endometriosis, were published during the years 2010–2011. Two studies targeted the Japanese population and one study concentrated on European women [26,27,28].

In the studies of the Japanese population, a relationship was demonstrated between rs10965235 polymorphism in the *CDKN2BAS* gene at *locus* 9p21 and rs16826658 polymorphism at the area of the *WNT4* gene at *locus* 1p36 [26].

An extensive GWAS research project was undertaken by the International EndoGene Consortium (IEC) in the population of British and Australian women with endometriosis [28]. Based on the conducted research, it was determined that 7p15.2 was the locus associated with the disease progression. This locus occurs among the so-far-identified genes that are responsible for the development of the uterus and the placenta [28].

The loci of 1p36 chromosome, revealed by the Japanese studies, had much less significance among the abovementioned populations [26].

In 2012, an international research team conducted what was then the largest association studies of the entire genome, the first one among women of the European origin, comparing DNA from 5586 women with endometriosis with 9331 women not suffering from the disease [29]. The team identified two regions of the genome associated with an increased risk of endometriosis. The first region is located at chromosome 7. This region may be involved in the control of nearby genes, which take part in the development of the uterus and its endometrium. The other variant is localized in proximity to *WNT4* gene, which participates in hormone metabolism and in the development and functioning of the female genital tract. The significant role of *WNT4* and *CDKN2BAS* and *FN1* was confirmed by studies of Pagliardini et al. [30].

Their studies indicate a correlation between 2p25.1 region, located close to the *GREB1* gene, and the risk of endometriosis [31].

The literature data on GWAS indicate that certain genetic variants, significantly more frequently occurring in endometriosis, seem to be good and promising functional candidates to play the role of genetic factors responsible for the emergence of the disease [32].

The research of GWAS in endometriosis is always continued, providing new results. The literature reports, concerning 998 Belgian patients with endometriosis and 783 controls, demonstrated that rs7521902, rs13394619, and rs6542095 polymorphisms could be related to endometriosis (Sapkota et al., 2015) [33]. It is noteworthy that three variants within *GREB1* area (close to rs13394619) and *CDKN2B-AS1* (close to rs1537377) also demonstrated nominally significant relationships with endometriosis [33].

It was suggested in the studies of Mafr et al. that an analysis of genetic variants at the area of the *WNT4* (rs3820282, rs16826658) gene could be helpful in the identification of patients from the group at high risk for the disease development [34]. 

Albertsen et al. conducted a GWAS study in a European population of 2019 cases of surgically confirmed endometriosis and 14,471 controls [35]. Three single nucleotide polymorphisms (SNPs) were associated with the disease: LINC00339-*WNT4* at *locus* 1p36.12 (rs2235529) and *RND3-RBM43* at *locus* 2q23.3 (rs1519761 and rs6757804). In the course of metanalysis, two more loci were identified as combined with endometriosis, namely, *RNF144B-ID4* on 6p22.3 (rs6907340) and *HNRNPA3P1*-LOC100130539 on 10q11.21 (rs10508881). 

In the study by the Polish team of Sobalska et al., a conducted GWAS analysis revealed statistically significant correlations between the new SNPs—not described in literature before—and endometriosis [36]. A relationship was observed in those studies between the rs10129516 polymorphism, located on chromosome 14 at the intergene—*PARP1P2* and *RHOJ*—area, and endometriosis. The *RHOJ* gene encodes one of many GTP (guanosin-5′-trifosphate)-binding small proteins of the Rho family, where GTP plays the function of energy carrier in the cell. Rho proteins dynamically control the assembly of components of the cytoskeleton in several physiological processes, such as cell proliferation and mobility. Rho proteins are also involved in pathophysiological processes, neoplastic transformation, and metastases. The protein, encoded by the *RHOJ* gene, is activated by the growth factor of the vascular endothelium and can control angiogenesis, as well as it plays an important role in the differentiation of adipocytes, endothelium mobility, and cytoskeleton formation [37]. Some overexpression of the *RHOJ* gene has been demonstrated in ectopic endometrium [38]. Endometriosis may be the cause of extrauterine (ectopic) pregnancy. The risk of such pregnancy is then increased in women suffering from endometriosis. It is, thus, possible to assume that the *RHOJ* gene can be involved in the development of endometriosis.

In the study of Nowaka et al., the authors found out that the prevalence of alleles of the *KIR* gene in patients with endometriosis did not differ from that in the control group [39]. In the case of the *KIR2DS5* gene, the HLA-C C2 + allele exerted a protective effect. It was suggested that the HLA-C C2 + allele could affect the ability of NK (Natural Killer) cells to eliminate ectopic endometrium in women who are its carriers. 

Studies of the Polish team of Sobalska et al. indicate the importance of rs644045 polymorphism, localized at *loci C2,* close to the *HLA-DRA* gene, for the development of endometriosis [36]. The *HLA-DRA* gene encodes the histocompatibility antigen, HLA of class II, the DR alpha chain, which plays a significant role in the immunological system. *Locus C2* is localized near the *HLA-DQA1* gene, in a distance below 200 kbp [40].

In early studies, conducted on the Chinese population, *HLA-DQA*1*0301 and *0401 alleles were associated with endometriosis [40]. The results, obtained by the Polish researchers, corresponded to the above observations and indicate that SNP polymorphisms near *C2* and *HLA-DRA* genes may be potential risk factors of endometriosis in Polish women.

The genetic analyses of Bylinska et al. confirmed the role of the *HLA-G* gene polymorphisms and of its *LILRB1* and *LILRB2* receptors for the development of endometriosis. HLA-G (the human leukocite antigen G) is recognized by KIR2DL4, LILRB1, and LILRB2 receptors on NK cells, the antigen presenting cells, lymphocyte T cells, and other cells. Some expression of HKA-G molecules was demonstrated in ectopic endometrium [41]. The genes for KIR2DL4, LILRB1, and LILRB2 receptors are polymorphic, which may influence their activity. The abovementioned Polish researchers analyzed if the polymorphisms of *HLA-G*, *KIR2DL4*, *LILRB1,* and *LILRB2* genes could affect the susceptibility to endometriosis and disease progression. It was demonstrated that the GG genotype of rs1632947 polymorphism of the *HLA-G* gene played a protective role, both against the disease and its severe stages; similarly, the CT genotype of rs1233334 polymorphism of the *HLA-G* gene protected against the disease progression. The AA genotypes of rs41308748 polymorphism of the *LILRB1* gene and the AG genotypes of rs383369 polymorphism of the *LILRB2* gene predisposed to endometriosis and its progression. No correlation was observed between the polymorphism of the KIR2DL4 gene and endometriosis [41].

In studies of another team of Polish researchers, a correlation was identified between rs12700667 and rs4141819 polymorphisms of the *RAF* gene and infertility in women with the advanced degree of endometriosis [42].

The latest studies indicate new genes and their polymorphisms that are associated with endometriosis. Christofolini et al. demonstrated a correlation of rs10928050 polymorphism of the *KAZN* gene and of rs2427284 polymorphism of the *LAMA5* gene with endometriosis [43]. 

Genetic studies provide sound evidence that changes in DNA increase—in some women—the probability of contracting endometriosis. The genetic contribution seems to be particularly large in the more serious forms of the disease. 

The long-running molecular studies of GWAS have resulted in an identification of a few genes—candidates to be the potential markers of endometriosis (Table 3) [44].

The to-date genetic analyses draw attention to the necessity of further knowledge expansion, regarding the discovered genetic polymorphisms and the incidence of certain diseases, taking into account considerable differences among the studied populations.

There is growing evidence that endometriosis is an inherited complex genetic feature, in which various genes, determining susceptibility to this disease, interact among themselves and with the environment to form a phenotype. The inherited susceptibility to contract endometriosis is then justified by the fact of the growing interest in the identification of genes and genetic polymorphisms, predisposing (qualifying) women to the group at risk for endometriosis development.

Literature data indicate the particular role of estrogen receptor genes, especially of the *ESR2* gene and of the *CYP19A1* gene for the susceptibility to and the incidence of endometriosis [61,62,63,64].

The estrogen receptors, which act as transcriptive factors, play a significant role in the growth and differentiation of endometrial cells, as well as in numerous biological functions in both, eutopic and ectopic endometrium. The ERβ receptor, encoded by the *ESR2* gene, is a dominating isoform in patients with endometriosis [65]. A different aromatase expression has been proven in endometrial foci, when compared to eutopic endometrium. *CYP19A1* is a gene which encodes aromatase—an enzyme participating in the biosynthesis of estrogens. The background of the observed changes is still far from being known and understood.

During the recent years, particular attention has been focused on the genetic mechanisms that may be of particular significance for the increased incidence rates of endometriosis. According to literature data, numerous studies are underway, seeking the markers of endometriosis. One of the ways is an evaluation of the expression levels of estrogen receptor genes and of the enzyme genes responsible for metabolism of drugs, including *ESR2* and *CYP19A1*, respectively.

## 3. The Role of the *ESR2* and *CYP19A1* Genes in Endometriosis

**Figure 1:**
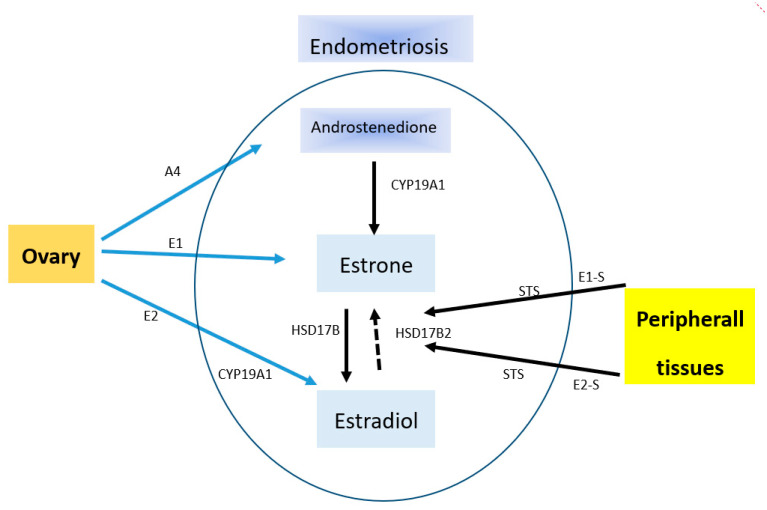
Source of estradiol in endometriosis. A4, androstenedione; CYP19A1, cytochrome P450 19A1 (aromatase); E1, estrone; E1-S, estrone sulfate; E2, estradiol; E2-S, estradiol sulfate; STS, steroid sulfatase; HSD17B, hydroxysteroid (17beta) dehydrogenase. Endometriosis is able to locally produce estrogens from E1-S and E2-S, which are circulating at high concentrations. This is due to the presence of steroid sulfatase activity. E2 can be inactivated through conversion to E1 by oxidative HSD17B activity. The major effects of estrogens are mediated by their classical nuclear receptors ESR 1 (Estrogen Receptor 1) and ESR2. CYP19A1 catalyzes the conversion of A4 to E1 as well as testosterone to E2.

Endometriosis is an estrogen-dependent disease. This confirms the fact of its occurrence in women of child-bearing age and of the remission of its symptoms after menopause or ovariectomy. This disease entity presents a specific ability to receive estrogen stimuli that stimulate its development. An enhanced local production of estrogens takes place within the endometrial tissue in result of an increased expression level of P450 cytochrome aromatase. Aromatase impacts the rate of estradiol synthesis (Figure 1). In addition, this phenomenon is also affected by deficits of 17β-hydroxysteroid dehydrogenase of type 2 (17β-HSD2), which is responsible for the oxidation of more active estradiol to less active estrone [65].

Genetic polymorphism occurs within the endometrial tissue, concerning estrogen and progesterone receptors [66,67], as well as the polymorphism of the enzymes responsible for the metabolism of drugs (*CYP1A1*, *CYP19*, and *GSTM1*), predisposing to endometriosis [68,69,70,71]. The abovementioned genetic variants may impact endometriosis in result of the increased production of estrogens under the effect of environmental contaminations, such as dioxins. Animal models demonstrated the participation of dioxins in causing endometriosis and estrogen-dependent tumors [72]. Literature data show that the risk of endometriosis is twice higher in patients with high levels of dioxins in blood serum [73].

The activation of estrogen receptors in endometriosis can be direct or indirect. The indirect activation is supported by *CYP1A1, CYP19,* and *GSTM1* polymorphic genes, which increase the level of P450 aromatase and estrogen production [74].

Estrone, 17β-estradiol (E2), and estriol belong to the estrogens produced in the human body. These are steroid hormones, produced from cholesterol in result of androgen aromatization under the influence of the enzymes from the P450 cytochrome group, localized in the internal mitochondrial membrane. The last, irreversible stage in estrogen production is demethylation, which occurs under the effect of p450 aromatase [75,76].

The function of estrogens in the genital system is:

— to stimulate the development of female genital organs;
— to form secondary and tertiary sexual characteristics;
— to stimulate proliferation of the stromal cells in endometrium and the growth of the uterine muscle mass, as well as the peristalsis of the uterus and of the ovarian ducts; and
— to stimulate the development of the breasts and of the external genital organs, in preparation of the breasts for lactation [77].

Estrogens reveal a relationship with the development of breast, prostate, uterine, ovarian, and endometrial cancer [62,78].

The level of estrogens in the body largely determines the activities of the enzymes that take part in their synthesis and catabolism, encoded, among others, by polymorphic genes. Therefore, studies are underway to reveal correlations between polymorphisms and estrogen-dependent diseases, the latter including breast cancer, endometrial cancer, osteoporosis, and, also, endometriosis [79,80,81]. The risk for the occurrence of endometriosis is evaluated in association with the polymorphisms of various genes, participating in the biosynthesis of estrogens, including *CYP19A1* and *ESR2*. 

The *CYP19A1* gene is localized on 15q21.2 chromosome, contains 10 exons, and encodes the aromatase of P450 cytochrome, the main component of the aromatase enzyme, which plays the most direct role in estrogen production [82].

The CYP19 aromatase converts testosterone and androstenedione into estradiol and estrone [83]. The foci of endometriosis, unlike eutopic endometrium, demonstrate the activity of aromatase. The presence of polymorphisms within the *CYP19A1* gene is associated with differences in the activity of aromatase. There are reports about possible correlations between this phenomenon and *CYP19A1* polymorphisms [84,85,86].

The *CYP19A1* gene is highly polymorphic. In its area (within intron 4), there is a polymorphism consisting in the occurrence of 7- to 12-fold tetranucleid repetitions of TTTA sequence with an additional, the shortest allele with seven repetitions of the sequence, together with the deletion of three TCT nucleotides above the microsatellite region [87,88].

In the case of endometriosis, it was studied together with 3pz I/D (insertion/deletion) polymorphism in intron 4. No correlation was found between TTTA repetitions and the incidence of endometriosis. In the case of 3pz I/D polymorphism, a weak but significant correlation was found with the incidence of the disease. Patients with endometriosis were most often the carriers of D/D genotype [89].

In the *CYP19A1* gene, besides the microsatellite polymorphism, there is also the SNP polymorphism in the noncoding region 3′, which is the transition of thymine into cytosine. It causes a decrease of the transcript stability [87,88].

Wang et al. reported that single nucleotide polymorphisms of *FSHR* (rs6165, rs6166), *HSD17B3* (rs2066479), and *CYP19* (rs700519) genes may modulate the risk of endometriosis in Taiwanese Chinese women [90]. The study showed that homozygous and heterozygous genotypes of four polymorphisms (rs6165 (GG + GA, 307Ala/Ala + 307Ala/Thr) of *FSHR*, rs6166 (GG + GA, 680Ser/Asn + 680Ser/Ser) of *FSHR*, rs2066479 (AA + AG, 289Ser/Ser + 289Ser/Gly) of *HSD17B3,* and rs700519 (TT + TC, 264Cys/Cys + 264Cys/Arg) of *CYP19)* were strongly associated with decreased risks of endometriosis [90]. 

In the studies of Vietri et al. a considerable prevalence was demonstrated of homozygotes A of Val89 polymorphism of the *CYP19A1* gene in women with endometriosis. This polymorphism may then play some role in the increase of the risk for endometriosis [91].

The latest research indicates a correlation of rs2899470 and 1531G  >  A of the *CYP19A1* gene with endometriosis [92,93].

However, not all reports confirm the correlation of SNP polymorphisms of the *CYP19A1* gene with endometriosis. The studies of Yi et al., while being a broad metanalysis, did not demonstrate any correlations between rs10046 polymorphism of the *CYP19A1* gene and the disease [63]. In another study by the Chinese researchers, rs2236722: T > C, rs700518: A > G, rs10046: T > C, and [TTTA]n polymorphisms of the *CYP19A1* gene were subject to a thorough analysis. Of all the studied genetic variants, only the rs700518AA genotype was associated with infertility in patients with endometriosis [94].

The highly polymorphic *CYP19A1* gene encodes aromatase, the activity of which was identified, besides normal tissues, in pathologically changed tissues, such as endometriosis, uterine fibromas, breast cancer, and endometrial cancer [95,96,97]. The *CYP19A1* gene demonstrates a tissue-specific expression and its regulation makes use of alternative promoters. 

The activity of estrogens on target cells is possible via estrogen receptors. Two types of receptors can be distinguished in the human body: the estrogen receptor α (ER α), encoded by the *ESR1* gene, and the estrogen receptor β (ERβ), encoded by the *ESR2* gene. 

ERβ is the main estrogen receptor in patients with endometriosis. Its presence is a significant and useful predictive factor. The modulation of gene transcription by estrogens is called the genomic activity of estrogens. The nongenomic mechanism of activity is characterized by a rapid response after an exposure to the hormone. The nongenomic system leads to post-translative modifications of signaling proteins. It is the ER receptor, localized in the cellular membrane, that is responsible for the nongenomic activity. Regarding this activity, neither the receptor binding to DNA nor the synthesis of gene mRNAs is needed.

The nongenomic activity of ERβ plays a dominating role in the progression of endometriosis [98].

The mechanism of the nongenomic activity of estrogens is very complex. Despite continued studies, it remains not fully exploited. The works, which are now conducted to learn the mechanism, turn toward studies on the integration of cellular signaling pathways, both on the surface and inside the mitochondria, which decide about cell apoptosis or survival. 

ERβ belongs to the family of nuclear receptors, the activation of which leads to receptor connection with a specific DNA sequence of the gene promoter, which may either activate or inhibit the transcription process. 

The genomic mechanism of estrogen activity involves the connection of estrogens to the receptor. Then, the complex is transferred to the cell nucleus. The receptor undergoes dimerization and binds to the estrogen response element (ERE). ERE is localized in the promoter of specific genes. In addition, the hormone binding induces conformational changes of the receptor in the ligand binding domain, which enables the connection of coactivator proteins [99].

The ERE sequence does not have more than one-third of human genes, the control of which takes place via the ER. The mechanism, by which estrogens control the transcription of these genes, remains unknown. It is known that estrogens regulate the expression of genes without binding to DNA, making use of the modulation of the functions of other transcriptive factor classes via the protein–protein interaction. The interaction of ER with AP-1, a known transcriptive factor, may be a good example. AP-1 (the activator protein 1) is a protein complex, built of protein dimers of Fos and Jun families.

A certain number of genes, which are controlled by estrogens and deprived of ERE, contain binding sites for the orphan nuclear hormone receptor SF-1 (SFRE) SF-1 response element, which bears the function of a direct binding site of the estrogen receptor (ERα) [99,100].

ERβ is encoded by the *ESR2* gene, localized on 14q23.2 chromosome [101,102,103].

The changes in ERβ biosynthesis usually precede the changes in the *ESR2* gene transcription and in mRNA levels. The gene variability may then affect the extent of the estrogen receptor biosynthesis [104].

In the case of *ESR2,* a correlation was found between the occurrence of menstrual disorders in women of the Chinese population with rs1256049 and rs4986938 polymorphisms [105]. A correlation was also identified in the Chinese women between rs17179740 A/G polymorphism of the *ESR2* gene and endometriosis [106].

The rs4986938 polymorphism of the ESR2 gene was also associated with advanced endometriosis observed in the Japanese population. No such correlation was confirmed in Greek, Italian, or Korean populations [107,108].

A team of Japanese researchers demonstrated that the specific polymorphism, which is observed in *ESR2* gene, is associated with the higher incidence of endometriosis in stage IV [109].

No specific marker for endometriosis has yet been identified. Groups of compounds are now searched, which, in a specific combination, would ensure a maximum sensitivity and specificity of non-invasive diagnostics of the disease, especially in its early stages [110,111]. The conducted broad assessment of the processes has recently been the subject of numerous analyses and studies. 

It has been proven that changes in the expression of genes and proteins, typical for ectopic foci of the disease, may also be found in the endometrium in the uterine cavity [112,113]. Many genes are subject to another expression in ectopic endometrium, when compared to normal, eutopic endometrium, which may play a key role in the development of endometriosis [114]. A different aromatase expression has been proven in endometriosis foci. A higher expression of P450 cytochrome and of hydroxysteroid 17-dehydrogenase in ectopic endometrium increases estrogen level, which activates the endothelial cells of the stroma and supports the development of endometriosis [61]. A significantly elevated level of the ERβ estrogen receptor and a decreased ERα level have been demonstrated in the tissue of endometriosis foci in comparison with the eutopic endometrium. 

## 4. The Expression of *ESR2* and *CYP19A1* Genes among Women with Endometriosis as a Potential Risk Factor for the Development of the Disease

The literature data on *ESR2* gene expression level in endometriosis are rather scarce. Such studies were undertaken by Osinski et al. [115], targeting infertility associated with endometriosis. Their study concentrated on the expression levels of hydroxy-delta-3-steroid dehydrogenase of type 2 (*HSD3B2*), hydroxysteroid 17-beta dehydrogenase of type 1 (*HSD17B1*) and of type 2 (*HSD17B2*), and the estrogen receptors of type 1 (*ESR1*) and of type 2 (*ESR2*), as well as of the androgen receptor (*AR*). The abovementioned genes are involved in metabolism and steroid activity in eutopic endometrium. The objective of that study was to examine whether infertility, associated with endometriosis, may result from disturbed expression of *HSD3B2, HSD17B1, HSD17B2,* and *ESR1/2* genes. The expression of the abovementioned genes was assayed in follicular phase and in luteal phase in women with preserved fertility and in patients suffering from endometriosis. Increased expression levels of *HSD3B2* gene and of *ESR1* gene were demonstrated in endometrium of infertile women with endometriosis. Those researchers drew a conclusion that it could have been associated with an abnormal activity of E2 estradiol on endometrium in that group of women.

According to the most recent studies, the following genes may account for the potential risk factors of infertility associated with endometriosis: *ESR1*, *ESR2*, beta hormone luteinizing gene, FOXP subunit, complement component 3 gene, and the Fc receptor-like 3 (*FCRL3*) gene [116,117,118,119]. It was recently suggested that *CYP17*, *VDR*, *MUC17*, *COX-2*, *WNT4*, *E-cadherin*, *CYP19*, *CYP17*, *NFKB1,* and *TYK2* genes, as well as the variants of *MUC2* gene, may also contribute to the risk of infertility associated with endometriosis [120,121,122,123,124,125].

Disorders in the level of *ESR2* gene expression, observed in endometriosis as related to the types and sites of lesions associated with the disease, are confirmed by world literature reports.

The team of Maekawa et al. employed the RT-PCR technique (real-time PCR) to assay the expression levels of *ESR1, ESR2,* and *PGR* genes in the cases of ovarian endometriosis and eutopic endometrium [126]. The *ESR1* and *PGR* expression levels were substantially lower, while the level of *ESR2* gene expression was significantly higher in endometriosis than in normal endometrium. DNA methylation levels were also studied. DNA methylation in *ESR1* gene was significantly higher in endometriosis than in eutopic endometrium. No major differences were observed in DNA methylation degrees for either *ESR2* or *PGR.* The researchers made a suggestion that the observed abnormal DNA methylation had been associated with a low level of *ESR1* expression, whereas the degree of methylation did not affect the expression changes of *ESR2* or *PGR* genes in endometriosis.

The latest studies of the team of Yilmaz et al. [64] demonstrated that the endometrial stromal cells, which mediate estrogen-induced inflammatory processes and the formation of prostaglandins, revealed an extremely low ratio of *ESR1* to *ESR2* expression levels, resulting from an excessive increase in the expression of the latter gene. The cells also demonstrated a deficit of the progesterone receptor, leading to resistance to progesterone and a defective retinoid synthesis. The expression of nuclear receptors, including the low levels of *ESR1* and *PGR* and a high level of *ESR2*, corresponded to the expression of the factors, which was found in the stem cells of uterine fibroid. It is suggested that the endometrial stromal cells may present certain features of the stem cells, present in other uterine tissues. In the study by Colon-Caraballo et al., [127] the expression levels of *ESR1*, *ESR2* estrogen, and *PGR* progesterone receptors were assayed in various types of endometrial changes and in eutopic endometrium of women with endometriosis and in a control group. Endometrial changes in the ovaries demonstrated low levels of *ESR1* and *PGR* expression and high levels of *ESR2* expression, while changes in the ovarian ducts were characterized by high expression levels of all the three receptors. Differences, observed among endometrial specimens, regarded lower *ESR1* expression and higher *ESR2* expression in the proliferative endometrium from the study group. A tendency toward the lack of nuclear activity of *PGR* was identified in the proliferative endometrium from the patients. The highest *ESR2:ESR1* expression ratio was observed in endometrial changes of the ovaries and in the excretory endometrium. The results, obtained by Colon-Caraballo et al., are valuable, as they may extend our knowledge regarding a potential, possible anticipation of individual reactions to hormonal therapies and provide a basis to develop personalized therapeutic methods for women with endometriosis.

The studies of Xue et al. [128] demonstrated that the levels of mRNA and of estrogen receptor 2 (ESR2) protein were significantly higher, whereas the levels of estrogen 1 receptor (ESR1), the total progesterone receptor (PGR), and of the progesterone B receptor (PGR B) were significantly lower in comparison with their corresponding values in normal endometrium. Since the *ESR2* gene expression level was characterized by exceptionally high differences between the cells of endometriotic lesions and the cells of normal endometrium, while the mechanisms of the difference remained unknown, the authors assumed a hypothesis that the change in DNA methylation process was the mechanism responsible for the elevated *ESR2* mRNA levels in the endometriotic cells. An area of CpG sites was localized at the promoter region (position –197/+ 359) of *ESR2* gene. A sequencing process in that region revealed a significantly higher degree of methylation in the primary cells of endometrium vs. that in the endometriotic cells. The applied demethylating agent (5-aza-2′-deoxycytidine) significantly increased *ESR2* mRNA levels in endometrial cells. It was demonstrated that the (−197/+ 372) region, which induced activity in the promoter, had also a CpG site and the *ESR2* promoter activity was strongly inactivated by methylation in vitro. Summing up, methylation of the CpG site at the *ESR2* promoter region was the main mechanism responsible for the differentiated expression of *ESR2* gene, both in endometriosis and in normal endometrium. 

Changes in the biosynthesis of the ERβ receptor were the consequence of the fluctuations in *ESR2* gene mRNA levels. A team of Yu et al. [129] assayed the expression levels of ERα, ERβ, TrkB, BDNF, and SGPL1 in patients with endometriosis, using the real-time PCR, Western blot, and immunohistochemical methods. A noticeable expression of ERα and SGPL1 occurred mainly in eutopic endometrium when compared to ectopic endometrium in patients with ovarian endometriosis, while the expression of ERβ, BDNF, and TrkB was mainly observed in ectopic endometrium of those patients. The expression of ERβ, ERα, TrkB, and SGPL1 concerned, first of all, eutopic endometrium in the proliferative phase, i.e., more than eutopic endometrium of the secretory phase. The authors suggested that an increased expression of ERβ in cytoplasm could mediate the pathogenesis of endometriosis.

The study by Matsuzaka et al. [130] indicated an increased expression of the estrogen receptor (ER-α) in active endometriosis (red lesions) vs. its non-active forms (brown and black or white endometriosis, corresponding to hemosiderin deposits or adhesions).

It was confirmed in the study by Xue et al., who suggested that differences in the ERα to ERβ ratio were more visible in various endometrial lesions. In particular, the ERα to ERβ expression ratio was much higher in active (red) endometriosis than in black endometriosis or in the case of endometrial cysts of the ovary [128]. 

Literature data demonstrate that the decreased ERα to ERβ expression ratio in endometrial changes may inhibit the expression of the progesterone receptor and contribute to resistance to hormonal therapy [131]. 

The latest data suggest that the farnesoid x receptor (FXR) may be a potential target for a future therapy in endometriosis [132]. It was shown that the activation of FXR (encoded by the *NR1H4* gene) inhibited the estrogen signaling pathway in breast cancer and testis tumors. A team of Wu et al. [132] conducted studies to explain if the activation of FXR by GW4064 substance, its synthetic agonist, may have a therapeutic influence on endometriosis and the molecular mechanisms in its background. It was demonstrated that FXR expression in endometriotic tissues and stromal cells was higher than in eutopic endometrial tissues and stromal cells. The dose-dependent approach to GW4064 cells led to a decrease in the expression of aromatase and the β estrogen receptor (ERβ) and induced the activation of ERK1/2 kinases, AMPK kinase, and the Stat3 transcriptive factor in the stroma. GW4064 significantly increased Stat3 phosphorylation, stimulating its binding to the *ESR2* promoter, which caused a decrease in ERβ level. Despite the carried-out studies, the significance of FXR for therapeutic purposes in endometriosis is, however, still poorly recognized and, thus, requires further research.

*CYP19A1* is a gene that encodes P450 cytochrome aromatase—an enzyme, participating in the biosynthesis of estrogens. A different aromatase expression has been proven in endometriosis foci [133]. The basis of the observed changes is not yet identified for the time being, therefore, the goals of the study included the demonstration of correlations between the level of the *CYP19A1* gene expression and the incidence of endometriosis. 

It is known that the control processes of the *CYP* genes’ expression in the cell are based on several mechanisms:

— The control of transcription of the p450 isoenzyme encoding genes,
— The mRNA stabilization, and
— Post-translative modifications.

In result of the transcription process induction, the transcriptive factors bind with DNA at the regulatory site, which is appropriate for them. The process is controlled by endo- or exogenous low-molecular ligands. By attaching to nuclear receptors, the ligands may, together with other specific proteins, lead to expression changes of the genes that are responsible for the metabolism catalyzed by P450 cytochromes [134].

It is known that the genetic polymorphism of *CYP1A1, CYP19*, and *GSTM1* enzymes, which are responsible for the metabolism of medicines, predisposes to endometriosis.

The activation of estrogen receptors in endometriosis can also be implemented indirectly via an abnormal activity of *CYP1A1*, *CYP19*, and *GSTM1* polymorphic enzymes, which increase the level of P450 aromatase and estrogen production [74].

Besides the analysis of *CYP1A1*, *CYP19,* and *GSTM1* genetic polymorphisms, the latest research concentrates on identifying the significance of the expression level of these genes in endometriosis.

Piccinato et al. showed that the expression of *CYP1A1* and *CYP1B1* gene mRNA was higher in endometriosis-related changes than in normal, eutopic endometrium [135]. The expression of the genes demonstrates also a certain dependence on the site where the changes occur. 

A reduced *CYP19A1* expression in endometriosis may result from epigenetic modifications in the regulatory regions of the gene as, for example, DNA methylation or from the modification of histones [136].

Changes in the methylation status of CpG islands, contained in *CYP19A1* gene, were confirmed in endometriosis by the team of Izawa et al. [137].

The CpG island is localized approximately 70 kb below exon 1.1 of the *CYP19A1* gene. It is hypomethylated in endometriosis (studies were run on stromal cells, obtained from chocolate ovarian cysts). However, its hypermethylation was found in eutopic endometrium (stromal cells obtained from women not suffering from endometriosis [138].

Van Kaam et al. demonstrated that methyl-cpg-binding proteins, such as MBD1 and MeCP2, which contain the methyl-CpG-binding N-terminal domain, and the transcription inhibition domain on the carboxyl terminus were not bound with the hypermethylated region of a CpG island in eutopic tissues [20]. It may explain the “muting” of aromatase expression in eutopic endometrium. The treatment of endometrial stromal cells with 5-aza-deoxycytidine (an irreversible inhibitor of DNA methyltransderase, which is necessary to maintain the status of genomic DNA methylation) induces the expression of aromatase. 

Demethylation of CpG islands in the *CYP19A1* gene may be significant for the control of aromatase levels [139], although other explanations are possible as well, since 5-aza-deoxycytidine changes the expression levels of a broad spectrum of genes, which may either indirectly or directly affect aromatase expression.

**Figure 2:**
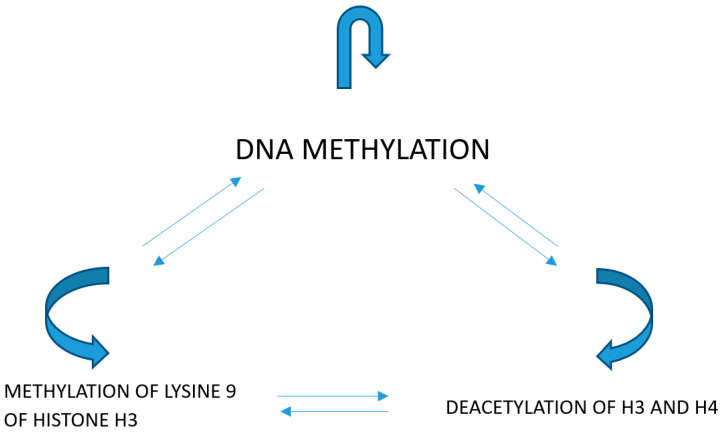
Methylation of lysine 9 of histone H3 causes the deacetylation of histones and methylation of DNA. DNA methylation and methylation of lysine 9 of histone H3 is a consequence of the deacetylation of histones. Methylation of DNA causes the deacetylation of histones and methylation of lysine 9 of histone H3.

Modifications of histones exert certain effects on the structure of chromatin and on the correlations of transcriptive factors with their corresponding response elements in gene promoters. The acetylation of H3 and H4 histones activates transcription by the relaxation of chromatin structure and enabling of the interaction of the transcriptive factors with their corresponding promoter elements. Lysine trimethylation at loci 9 and 27 within H3 histone inactivates the transcription, causing a higher condensation of chromatin (Figure 2).

In the studies performed on rat granulosa cells it was demonstrated that modifications of histones affected the expression of *CYP19A1* mRNA [94].

Ishikawa et al. [140] stated that the acetylation of H3 and H4 at the region of I.4 promoter by dexametazone occurred during the induction of aromatase expression in the cell lines of breast cancer. No modifications of histones were noted with regards to aromatase regulation in endometriosis.

The inhibition of aromatase mRNA transcription by microRNA was described in tissues of mammalian ovaries (miR-503 and miR-378, direct activity; miR-224 and miR-383, indirect activity), in the differentiation of trophoblasts (miR-19b and miR-106a), and in endometrial cancer (miR98) [141]. There are no reports on the control of aromatase expression by microRNAs in endometriosis.

The latest studies indicate that the overexpression of miR-370-3p inhibits cell proliferation and induces apoptosis in endometrial cells [142]. The results show that miR-370-3p can play the role of a negative regulator of the steroidogenic factor 1 (SF-1), influencing in this way the proliferation of endometrial cells.

It has been shown that a decreased level of miR-23a and b in endometrium of women with endometriosis releases an elevated expression level of SF-1, as well as of the steroidogenic acute regulatory protein (StAR) and of CYP19. It suggests some role of miR-23a and b in the acquisition of steroidogenic activity by endometrial tissues [143].

A team of Maia et al. [22] obtained results, clearly indicating that *CYP19A1* expression might not be correlated with endometriosis. The researchers conducted their studies to see whether the expression of aromatase in eutopic endometrium correlated with the presence of endometriosis and its clinical staging in patients with infertility and/or dysmenorrhea. The patients were submitted to laparoscopy and hysteroscopy. The study involved 106 women in the reproductive age and with symptoms of dysmenorrhea and infertility. A control group consisted of 16 patients with asymptomatic endometriosis. The results of the studies demonstrated that aromatase expression in the endometrium was connected with the occurrence of dysmenorrhea and infertility, regardless of whether endometriosis was present or not. 

The results of experimental study, concerning the analysis of *ESR1*, *ESR2*, *PGR,* and *CYP19A1* genes, unveil the existence of certain correlations of their expression with endometriosis. A significant increase of *ESR2* and *CYP19A1* gene expression was observed in the studied groups of women with endometriosis, which may indicate a certain role of this factor in the pathogenesis of endometriosis. The results, obtained in experimental study, contribute to better knowledge of and information on the molecular mechanisms that support the development of endometriosis. In consideration of the rather small number of studies on the expression and *CYP19A1* and *ESR2* gene in patients with endometriosis, the assumed impact of experimental studies may bring a real added value, extending our knowledge of the effects of genetic factors on the development of endometriosis. Learning and understanding the relationships between *ESR2* and *CYP19A1* gene expression and endometriosis may help design and develop new therapeutic concepts and strategies in the context of the disease. Studies on gene expression aspects are apt to constitute a prima facie target of major concern, regarding personalized therapy.

## 5. Conclusive Summary

While reviewing the studies on endometriosis from recent years, it is becoming apparent that the currently available therapeutic measures paint a rather bleak picture, not translating into a bright future for the patients. Regrettably, the available treatment protocols of this disease remain still ineffective. Nevertheless, the continuous progress of knowledge, especially at the genetic level, which has been made over recent years, allows the identification of certain molecular targets for new therapeutic methods. Great hopes are associated with new research trends, as, for example, the application of miRNA or lncRNA, which controls the key cell pathways in the development of the disease, as molecular markers in endometriosis [144]. However, the studies have to encompass large groups of patients, together with their full clinical descriptions, in order to draw conclusions regarding the use of the molecules as endometriosis markers. There are reports that inform about the development of new application possibilities for ncRNAs as diagnostic tools [25]. We can only hope that genetic studies will enable setting up new therapeutic protocols and significantly contribute to a major improvement of therapeutic outcomes in women suffering from endometriosis.

## Figures and Tables

**Table 1:** MicroRNAs (miRNAs) involved in the development of endometriosis.

Biological Process in Cells	miRNA Type
Hypoxia	MiR-20a, miR-148a
Survival and proliferation	miR-10b, miR-29c, miR-100, miR-143, miR-145, miR-183, miR-202, miR-210, miR-451
The inflammatory state	miR-302a, miR-542-3p
Steroidogenesis	miR-142-3p, miR-23, miR-135
Reconstruction and angiogenesis	miR-21, miR-93, miR-199a-5p, miR-210, miR-520g

**Table 2:** Long noncoding RNA (lncRNA) expression disorders in endometriosis.

IncRNA Type					
Expression Increase			Expression Decrease		
Plasma ^a^	Ectopic Endometrium in the Ovary ^b^	Eutopic Endometrium ^c^	Plasma ^a^	Ectopic Endometrium in the Ovary ^b^	Eutopic Endometrium ^c^
NAPA-AS1-202 RAB-203	CHL-AS2 LOC255167 LOC400043 XLOC_012904 XLOC_12_009510 AFAP1-AS1	AC068282.3 XLOC_004134 GBP1P1 RP11-369C8.1 AX746484 BC025370	NIT2-203 PEF1-206 MTMR11-206 CCDC91-218 POLD4-204	LOC100505776 UCA1 XLOC_012981 LINC00261 LOC100507043 LOC440335	RP11-403H13.1 RP11-679C8.2 RP11-77A13.1 RP11-408H2.0.1 CHRM3-AS2 AC007246.3

^a^ Plasma of women with endometriosis vs. healthy women. ^b^ Ectopic endometrium of the ovaries vs. eutopic endometrium of women with endometriosis. ^c^ Eutopic endometrium of women with endometriosis vs. healthy women.

**Table 3:** Candidate genes associated with endometriosis development.

Gene	Function
*WNT4*	The gene encoding a protein which is necessary for the development of the female genital tract. In mice, deprived of the *WNT4* gene, there is almost a complete lack of the Müllerian ducts and their derivatives [45]. *WNT* expression was shown in the peritoneum and endometrium [46].
*GREB1*	The gene of early response on the pathway of oestrogen regulation, which is involved in the hormone-dependent growth of breast cancer cells. Its expression grows in endometriotic lesions of the peritoneum, in comparison with eutopic endometrium. It plays some role in the oestrogen-dependent development of endometriosis [47].
*ETAA1*	It encodes the surface antigen, specific for the neoplasms of the Ewing’s sarcoma family (a group of neoplasms which are formed in the bone or in the soft tissue and develop from the same type of stem cells in the body [48].
*FN1*	It is involved in the processes of adhesion and migration of cells, including embryogenesis, wound healing processes, blood coagulation, host protection and metastases [49]. It has been demonstrated that the *SOX2* gene, which is a transcriptive factor for *FN1*, controls the migration of cells in ovarian cancer [50]
*ID4*	The ovarian oncogene which participates in control of the methylation pathway during breast cancer formation [51]. Its elevated expression occurs in the ovary and in the cell lines of endometrial cancer and breast cancer [52].
*NFE2L3*	A transcriptive factor, the contribution of which is suggested in the process of differentiation of cells, the inflammatory state and carcinogenesis [53]. Its enhanced expression has been demonstrated in the human cells of breast cance [54] and in tissue samples of testis cancer [55].
*miRNA_148a*	This microRNA takes part in the signalling pathway of Wnt/β-catenin whoch may play some role in the development of endometriosis via homeostasis regulation by the sex hormone [56] and fibrogenesis [57].
*HOX A10*	It belongs to the homeobox family of transcription factors, which plays an important role in the differentiation of the Müllerian duct to the ovarian duct, the uterus, the uterine cervix and the vagina [58].
*CDKN2B-AS1*	It is involved in the regulation of the suppressor genes: *CDKN2B*, *CDKN2A* and *ARF*. *CDKN2A* inactivation has been described in endometriosis and endometrial cancer [59].
*VEZT*	It encodes a protein which is the main component of the cadherin-catenin complex, necessary to create and maintain the adherens junctions. This protein undergoes expression in the majority of epithelial cells and is of key importance for the formation of cell junctions. It is also an important suppressor gene, impacting the genes associated with migration and invasion of cells, the growth genes, the genes of adhesive cells and the *TCF19* gene. *TCF19* is a potential transactivating factor which may play an important role in the transcription of genes required for the later stages of cell cycle progression. *TCF19* takes part in keeping the immunological balance [60].
